# Differing roles of autophagy in HIV-associated neurocognitive impairment and encephalitis with implications for morphine co-exposure

**DOI:** 10.3389/fmicb.2015.00653

**Published:** 2015-07-06

**Authors:** Seth M. Dever, Myosotys Rodriguez, Jessica Lapierre, Blair N. Costin, Nazira El-Hage

**Affiliations:** ^1^Department of Pharmacology and Toxicology, School of Medicine, Virginia Commonwealth University, RichmondVA, USA; ^2^Department of Immunology, Herbert Wertheim College of Medicine, Florida International University, MiamiFL, USA

**Keywords:** autophagy, HIV-associated neurocognitive disorders, HIV encephalitis, microarray, microglia, neuron, morphine

## Abstract

We investigated the role of autophagy in HIV-infected subjects with neurocognitive impairment (NCI) ± HIV encephalitis (HIVE), many of which had a history of polysubstance abuse/dependence, using post-mortem brain tissues to determine whether differences in autophagy related factors may be more associated with NCI or NCI-encephalitis. Using qRT-PCR, we detected significant differences in gene expression levels with *SQSTM1, LAMP1* higher in HIV-infected subjects without NCI while *ATG5, SQSTM1* were then lower in HIV infection/NCI and *ATG7, SQSTM1* being higher in NCI-HIVE. Immunohistochemical labeling of these autophagy associated proteins (also including Beclin 1 and LC3B) in Iba1-positive microglial cells showed generally higher immunoreactivity in the NCI and NCI-HIVE groups with more focal vs. diffuse patterns of expression in the NCI-HIVE group. Furthermore, analysis of microarray data from these same subjects found significantly higher levels of *LAMP1* in NCI-HIVE compared to uninfected subjects in the basal ganglia. Finally, we tested the effect of supernatant from HIV-1-infected microglia and HIV-1 Tat protein in combination with morphine on neurons *in vitro* and found opposing events with both significant inhibition of autophagic flux and reduced dendrite length for morphine and supernatant treatment while Tat and morphine exposure resulted in lower autophagic activity at an earlier time point and higher levels in the later. These results suggest autophagy genes and their corresponding proteins may be differentially regulated at the transcriptional, translational, and post-translational levels in the brain during various stages of the HIV disease and that infected individuals exposed to morphine can experience mixed signaling of autophagic activity which could lead to more severe NCI than those without opioid use.

## Introduction

The central nervous system (CNS) is preferentially vulnerable to human immunodeficiency virus (HIV) infection as chronic exposure to HIV in the brain can lead to neurocognitve impairment (NCI) from HIV-associated neurocognitive disorders (HANDs; Nabha et al., 2013). HAND can range from asymptomatic neurocognitive impairment (ANI) intermediately to HIV-associated mild neurocognitive disorder [MND; formerly referred to as minor cognitive and motor disorder (MCMD)] to the most severe condition of HIV-associated dementia (HAD; Antinori et al., 2007; Woods et al., 2009), manifested pathologically as HIV encephalitis (HIVE; Zink et al., 1999), through interactive cellular events (Kaul et al., 2005). As neurons have not generally been found to be infected by HIV (Kramer-Hammerle et al., 2005; Verma et al., 2010), most of the HIV-associated neurotoxicity can occur from bystander effects through the actions of infected/activated glia (Kaul et al., 2001). Microglia are the primary target of HIV in the brain (Kramer-Hammerle et al., 2005), and this cell type can release various toxic and inflammatory factors during the course of infection resulting in neuronal injury leading to HAD (Anderson et al., 2002; Garden, 2002; Gonzalez-Scarano and Martin-Garcia, 2005).

Macroautophagy (hereafter referred to as autophagy) is a critical mechanism to ensure proper homeostasis and functioning of the cell through removal of unused and damaged cellular organelles and components. In the process of autophagy, autophagosomes carrying these contents fuse with lysosomes creating post-fusion autolysosomes during autophagic flux in which the contained cellular material is degraded and recycled (Klionsky and Emr, 2000). Although disruptions to autophagy in neuronal cells have been implicated in neurodegenerative diseases (Komatsu et al., 2007a; Cherra and Chu, 2008), less is known about the role of autophagy in microglia and how modulation of autophagic processes in microglial cells may intercellularly lead to alterations of this and other pathways in bystander cell types such as neurons, particularly in the context of HIV infection. While autophagy may be thought of as being a generally cytoprotective process in response to various factors (Moreau et al., 2010), it appears to play a complex role in viral replication with cells permissive to productive HIV infection being both induced (Wang et al., 2012), and inhibited (Zhou and Spector, 2008), which may be selectively occurring to promote virus production at different stages of the viral life cycle (Espert et al., 2009). However, initial compensatory higher levels of autophagic activity in bystander cells such as neurons to remove viral-induced toxins followed by cellular differences resulting in reduced or defective autophagy may lead to neurodegeneration and neurocognitive deficits if an accumulation of protein aggregates results from the process not being driven to completion (Gelman and Schuenke, 2004; Yu et al., 2005).

The autophagic machinery consists of a variety of autophagy-related (*ATG*) genes and their encoded protein products (ATG; Klionsky, 2012). Beclin 1 (ATG6) is involved in the initiation of autophagosome formation (Liang et al., 1999), while the yeast Atg8 mammalian homolog LC3, of which LC3B is the most commonly examined isoform (Mizushima et al., 2010), associates with the autophagosomal membrane to promote its elongation and maturation following phosphatidylethanolamine conjugation in the conversion of LC3-I to the lipidated form LC3-II through ubiquitin-like reactions (Kabeya et al., 2000, 2004). ATG7 acts as an E1-like enzyme involved in formation of the ATG12–ATG5 conjugate (Tanida et al., 2001, 2012), which then acts as an E3-like enzyme in the LC3 lipidation reaction (Hanada et al., 2007). In addition to components directly involved in the autophagic process, markers of autophagic activity have also been identified. Sequestosome 1 (p62/SQSTM1) binds to Atg8/LC3 and disruptions in autophagy have been found to be associated with higher p62/SQSTM1 expression levels (Bjorkoy et al., 2005; Wang et al., 2006; Komatsu et al., 2007b; Pankiv et al., 2007), while higher lysosomal-associated membrane protein 1 (LAMP1) can indicate the presence of more lysosomes and autophagosomes during rises in autophagic activity (Klionsky et al., 2008).

While cellular differences resulting from prolonged HIV infection can result in NCI, drugs of abuse such as opiates can exacerbate and accelerate the onset of these deficits (Hauser et al., 2012). HIV/AIDS and opiate drug abuse are interlinked epidemics among injection drug users (Leshner, 1998), and morphine, the main bioactive product of heroin in the brain (Wright, 1940; Jaffe and Martin, 1985), can enhance HIV-1 replication (Steele et al., 2003), as well as neuronal toxicity in the presence of HIV-1 Tat and gp120 proteins (Zou et al., 2011; Podhaizer et al., 2012). However, whether alterations to autophagy may be a contributing factor in these interactive effects is largely unknown.

In the present study, we examined the role of autophagy in HIV-associated NCI using post-mortem brain tissues from HIV-infected subjects with NCI ± HIVE as well as unimpaired infected subjects, many of which had a history of polysubstance abuse/dependence, to determine whether the differences that we found were more associated with impairment or encephalitis. We also performed *in vitro* studies to examine the effect of HIV-morphine interactions on autophagy in primary human neurons. The findings suggest mixed autophagic signals in the HIV-infected brain may be a mechanism resulting in cellular dysfunction leading to NCI that is more associated with HIVE than NCI alone. Additionally, supernatant from HIV-1-infected primary human microglia and HIV-1 Tat protein in combination with morphine can modify the autophagic activity of neurons in opposing ways which could accelerate the onset of these deficits.

## Materials and Methods

### Human Brain Tissue

Human brain tissue was obtained from a subset of samples used in the National NeuroAIDS Tissue Consortium (NNTC) Gene Array Project (Morgello et al., 2001; Gelman et al., 2012). Briefly, the array project consists of four groups of subjects [HIV-negative (Group A), *n* = 6; HIV-positive (Group B), *n* = 6; HIV-positive with neurocognitive impairment (NCI; Group C), *n* = 7; and HIV-positive with combined NCI and HIVE (Group D), *n* = 5] with post-mortem tissue samples taken from three brain regions across the groups [the frontal lobe white matter, *n* = 9; frontal cortex, *n* = 10; and basal ganglia, *n* = 1 (or combined frontal lobe white matter/frontal cortex tissue, *n* = 8), where *n* = the total number of samples obtained for all groups combined]. Details on the specific brain regions and numbers of individual samples analyzed for each subject group in this study as well as age, neurocognitive diagnosis, brain pathology, and substance use histories for each subject are listed in **Table 1** and have been described previously (Dever et al., 2012, 2014).

**Table 1:** Sample origins, neurocognitive diagnoses, and substance use histories of subjects.

ID^a^	Age	Sample origin	Neurocognitive diagnosis^b^	Brain pathology^c^	PRISM/CIDI substance use history^d^
A1	44	Frontal lobe white matter	Normal	No path	Not assessed
A2	53	Frontal lobe white matter	Normal	No path	Not assessed
A3	63	Frontal lobe white matter	Normal	No path	Not assessed
A4	58	Combined frontal lobe white matter/frontal cortex	Normal	No path	Not assessed
A5	34	Combined frontal lobe white matter/frontal cortex	Normal	No path	Not assessed
A6	48	Frontal cortex	Normal	No path	Not assessed
B1	49	Frontal lobe white matter	Normal	No path	Cannabis dependence, opiate dependence
B2	47	Combined frontal lobe white matter/frontal cortex	Normal	No path	Alcohol abuse/dependence, cannabis abuse/dependence, cocaine abuse, hallucinogen abuse, opiate abuse, sedative abuse, stimulant abuse
B3	44	Combined frontal lobe white matter/frontal cortex	Normal	No path	No history reported
B4	59	Frontal cortex	Subsyndromic	No path	Alcohol abuse
B5	60	Frontal cortex	Normal	No path	Cannabis abuse, cocaine abuse/dependence, hallucinogen abuse, stimulant abuse/dependence, other drug abuse/dependence
B6	39	Frontal lobe white matter Frontal cortex	Normal	Minimal non-diagnostic abnormalities	No history reported
C1	33	Frontal lobe white matter	“Possible HAD”	Minimal non-diagnostic abnormalities	Cannabis dependence, cocaine dependence
C2	35	Combined frontal lobe white matter/frontal cortex	MCMD	Alzheimer’s type 2 gliosis, focal infarct	No history reported
C3	57	Combined frontal lobe white matter/frontal cortex	“Possible HAD”	Atherosclerosis of brain	Alcohol abuse/dependence, stimulant abuse/dependence
C4	36	Frontal cortex	MCMD	Lymphoma	Not assessed
C5	52	Frontal cortex	MCMD	No path	Alcohol abuse/dependence
C6	41	Frontal lobe white matter Frontal cortex	“Probable HAD”	Minimal non-diagnostic abnormalities	Not assessed
C7	52	Frontal lobe white matter Frontal cortex Basal ganglia	HAD	Other non-infectious path	Alcohol abuse/dependence, hallucinogen abuse, sedative abuse, stimulant abuse/dependence, other drug abuse
D1	48	Frontal lobe white matter	“Probable MCMD”	HIVE	Cocaine dependence, sedative dependence
D2	32	Combined frontal lobe white matter/frontal cortex	HAD	HIVE, microglial nodule encephalitis	Alcohol abuse/dependence, opiate abuse/dependence, sedative abuse/dependence
D3	39	Combined frontal lobe white matter/frontal cortex	HAD	No path	No history reported
D4	55	Frontal cortex	HAD	HIVE	Not assessed
D5	41	Frontal cortex	“Probable HAD”	CMV encephalitis, HIVE, lymphoma, microglial nodule encephalitis, other infections, PML	Not assessed

*^a^Group A subjects, HIV-negative; Group B, HIV-positive; Group C, HIV-positive/neurocognitively impaired; Group D, HIV-positive/neurocognitively impaired with HIV encephalitis (HIVE).*

*^b^Subsyndromic, mildy abnormal test performance without symptoms of impairment; HAD, HIV-associated dementia; MCMD, minor cognitive and motor disorder.*

*^c^Following subject selection, D5 was reported to have brain co-infection with cytomegalovirus and papovavirus but had array results similar to the other subjects in Group D on a principal components analysis and therefore remained included in this and the original study (Gelman et al., 2012).*

*^d^PRISM, Psychiatric Research Interview for Substance and Mental Disorders; CIDI, Composite International Diagnostic Interview.*

### Quantitative Real Time-Polymerase Chain Reaction

Total RNA was isolated using the miRNeasy Mini Kit (Qiagen; Valencia, CA, USA) and used to generate cDNA templates by reverse transcription of 1 μg RNA using the High Capacity cDNA Reverse Transcription Kit (Applied Biosystems; Carlsbad, CA, USA) according to the manufacturer’s instructions. PCR reactions were performed in a total volume of 20 μL containing SensiMix SYBR qPCR reagents (Bioline; Tauton, MA, USA) using a Corbett Rotor-Gene 6000 real-time PCR system (Qiagen). PCR conditions consisted of an initial hold step at 95°C for 10 min followed by 40 amplification cycles of 95°C for 10 s, 58°C for 30 s, and 72°C for 30 s. Sequences of the primer sets used are listed in **Table 2**. The specificity of the amplified products was verified by melting curve analysis and agarose gel electrophoresis. qRT-PCR data were calculated as relative expression levels by normalization to GAPDH mRNA using the 2^-ΔΔCt^ method (Livak and Schmittgen, 2001).

**Table 2:** Primer sets used for qRT-PCR.

Gene	Forward primer	Reverse primer
*BECN1*	5’- GCTGAGAGACTGGATCAGGA -3’	5’- ATTGTGCCAAACTGTCCACT -3’
*MAP1LC3B*	5’- GAATTCTCCCACACCAAGTG -3’	5’- AAATAGTGAACCCCATGCAA -3’
*ATG7*	5’- TGTGTTGGAGATTGGTTCCT -3’	5’- GAGATCTTGGCATTGTCCAC -3’
*ATG5*	5’- ATGCAGGGAACACTAAGCTG -3’	5’- TCTAGGGCATTGTAGGCTTG -3’
*SQSTM1*	5’- GAGTTCCAGCACAGAGGAGA -3’	5’- AAGACAGATGGGTCCAGTCA -3’
*LAMP1*	5’- AGTGTCTGCTGGACGAGAAC -3’	5’- GACCCTAAGCCCAGAGAAAG -3’
*GAPDH*	5’- CATGGCACCGTCAAGGCTGAGAA -3’	5’- CAGTGGACTCCACGACGTACTCA -3’

### Immunohistochemistry

Frozen brain tissue from the NNTC Gene Array Project subjects was sectioned to 5 micron thickness, fixed in 4% paraformaldehyde, permeabilized with 0.5% Triton X-100, blocked in 10% milk/0.1% goat serum, and immunolabeled. Primary antibodies used were anti-p62/SQSTM1 (catalog number NBP1-48320) at a 1:100 dilution and anti-Beclin 1 (catalog number NB500-249), anti-LC3B (catalog number NB600-1384), anti-ATG5 (catalog number NB110-53818), and anti-LAMP1 (catalog number NB120-19294) at a 1:400 dilution from Novus Biologicals (Littleton, CO, USA); anti-APG7/ATG7 (Santa Cruz Biotechnology; Santa Cruz, CA, USA; catalog number sc-33211) at a 1:100 dilution; and anti-Iba1 (Abcam; Cambridge, MA, USA; catalog number ab5076) at a 1:100 dilution. Immunoreactivity was visualized with secondary antibodies from Molecular Probes (Carlsbad, CA, USA) conjugated to Alexa Fluor 488 dye (catalog number A11055) for Iba1 and Alexa Fluor 594 dye (catalog number A11037) for autophagy proteins and related markers, both used at a 1:200 dilution. DAPI staining was used to label cell nuclei.

### Microarray Data Analysis

National NeuroAIDS Tissue Consortium Gene Array Project CEL files for individual arrays were retrieved from the NCBI Gene Expression Omnibus^1^, GEO accession number GSE35864 (Gelman et al., 2012), and re-analyzed as described previously (Dever et al., 2014). Briefly, robust multi-array average (RMA) analysis for probe intensity data normalization (Irizarry et al., 2003), and multi-class linear models for microarray data (Limma) analysis to access differential expression between the subject groups (Smyth, 2004), were performed for each brain region. Limma output *p*-values were adjusted by Benjamini and Hochberg’s (1995) false discovery rate correction for multiple testing, setting the significance level to 0.05. Heat maps were constructed from probeset RMA values using MultiExperiment Viewer 4.9^2^ (Saeed et al., 2003, 2006).

### Preparation of Supernatant from HIV-1-Infected Microglia

Primary human microglia were purchased from ScienCell Research Laboratories (Carlsbad, CA, USA; catalog number 1900-f1) and cultured according to the manufacturer’s instructions. Cells were infected with the macrophage-tropic HIV-1 SF_162_ strain [obtained through the NIH AIDS Reagent Program, Division of AIDS, NIAID, NIH: HIV-1 SF_162_ from Dr. Jay Levy (Cheng-Mayer and Levy, 1988)] at a concentration of 50 pg HIV-1 p24/10^6^ cells as performed previously (El-Hage et al., 2013, 2014, 2015). Seven days post-infection, the cell culture supernatant was collected and passed through a 0.20 μm filter. Productive infection was confirmed and HIV-1 p24 protein levels were measured in supernatants using the HIV-1 p24 Antigen Capture Assay (Advanced Bioscience Laboratories; Rockville, MD, USA; catalog number 5421).

### Neuronal Cell Treatment and Immunocytochemistry

Primary human neurons were purchased from ScienCell Research Laboratories (catalog number 1520) and cultured according to the manufacturer’s instructions. Cells were treated with supernatants from uninfected or HIV-1-infected (30 pg/mL HIV-1 p24) microglia and 500 nM morphine sulfate (Sigma-Aldrich; St. Louis, MO, USA; catalog number M8777) alone or in combination for 24 h and fixed in 4% paraformaldehyde, permeabilized with 0.5% Triton X-100, blocked in 10% milk/0.1% goat serum, and immunolabeled. Primary antibodies were anti-p62/SQSTM1 (Novus Biologicals; catalog number NBP1-48320) and anti-MAP2 (Abcam; catalog number ab5392), both used at a 1:100 dilution. Immunoreactivity was visualized with secondary antibodies from Molecular Probes conjugated to Alexa Fluor 594 dye (catalog number A11037) for p62/SQSTM1 and Alexa Fluor 488 dye (catalog number A11039) for MAP2, both used at a 1:500 dilution. DAPI staining was used to label cell nuclei.

### Neuronal Cell Transfection and Treatment

Neurons were transfected with the mRFP-GFP-LC3 tandem fluorescently tagged LC3 plasmid ptfLC3 [Addgene; Cambridge, MA, USA; catalog number 21074 (Kimura et al., 2007)] using Lipofectamine 2000 reagent (Invitrogen; Carlsbad, CA, USA). Fourty-eight hours post-transfection, cells were treated with 100 nM HIV-1 IIIB recombinant Tat_1-86_ protein (ImmunoDX; Woburn, MA, USA; catalog number 1002-2) and 500 nM morphine alone or in combination for 8 h. Cells were fixed in 3.7% paraformaldehyde and stained with DAPI to label cell nuclei.

### Confocal Microscopy and Image Analysis

A Zeiss LSM 700 confocal laser scanning microscope (Carl Zeiss; Thornwood, NY, USA) was used to image tissue sections with a 63x oil immersion objective and transfected neurons at 20x magnification. Neurons treated with microglial supernatants were imaged using a Zeiss LSM 710 confocal laser scanning microscope (Carl Zeiss) at 20x magnification. Images were collected using ZEN 2011 (blue edition) software (Carl Zeiss) and edited with Adobe Photoshop CS3 Extended 10.0 (Adobe Systems; San Jose, CA, USA). Relative levels of immunofluorescent intensity were quantified using plugin algorithm software for ImageJ [National Institutes of Health (NIH); Bethesda, MD, USA]. Dendrite length was measured by tracing along individual neurons fluorescently labeled with MAP2 antibody using the broken line tool in ImageJ (Smith et al., 2009).

### Western Blotting

Cell lysates were prepared in RIPA buffer supplemented with a mixture of protease and phosphatase inhibitors following 24 h treatment and separated by SDS-PAGE for immunoblotting. Primary antibodies used were anti-LAMP1 (catalog number NB120-19294) at a 1:100 dilution, anti-Beclin 1 (catalog number NB500-249) and anti-ATG5 (catalog number NB110-53818) at a 1:500 dilution, and anti-LC3B (catalog number NB600-1384) and anti-p62/SQSTM1 (catalog number NBP1-48320) at a 1:1000 dilution from Novus Biologicals (Littleton, CO, USA); anti-APG7/ATG7 (Santa Cruz Biotechnology; Santa Cruz, CA, USA; catalog number sc-33211) at a 1:50 dilution; and anti-GAPDH (Sigma-Aldrich; St. Louis, MO, USA; catalog number G9545) at a 1:1000 dilution. Primary antibodies were followed by incubation a secondary antibody conjugated to horseradish peroxidase (Cell Signaling Technology; Danvers, MA, USA; catalog number 7074) used at a 1:1000 dilution. Immunoblots were exposed to SuperSignal West Femto Substrate (Thermo Scientific; Waltham, MA, USA) and visualized using a ChemiDoc imaging system (Bio-Rad; Hercules, CA, USA).

### Statistics

Data were analyzed by one-way ANOVA followed with Student Neuman-Keuls *post hoc* test for multiple comparisons using GraphPad Prism 5 (GraphPad Software; La Jolla, CA, USA). A value of *p* < 0.05 was considered significant. Outliers were removed from the qRT-PCR analysis and identified with Grubbs’ test using the QuickCalc outlier calculator (GraphPad Software), setting the alpha level to 0.05.

## Results

### Differential Expression of Autophagy Associated Genes in HIV-Infected Subjects with Two Types of Neurocognitive Impairment

**FIGURE 1:**
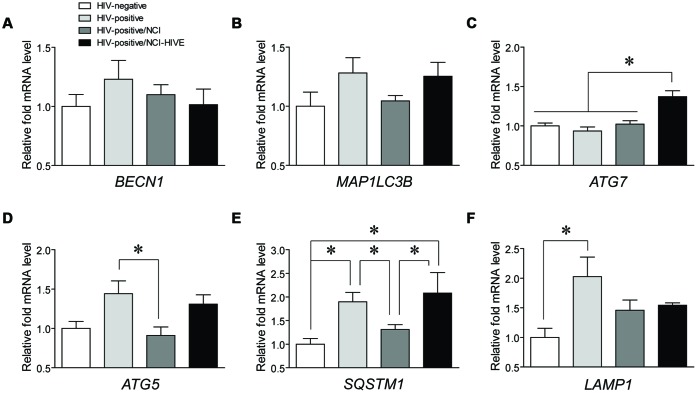
**Expression of mRNAs from autophagy associated genes in HIV-infected subjects with varying levels of neurocognitive impairment.** Expression levels of the autophagy genes **(A)** *BECN1*, **(B)** *MAP1LC3B*, **(C)** *ATG7*, and **(D)** *ATG5*, and autophagic activity markers **(E)** *SQSTM1* and **(F)** *LAMP1* were measured by qRT-PCR across the indicated subject groups. Data are presented relative to HIV-negative subjects which was set to a value of 1. *BECN1*: *F*(3,24) = 0.7201, *p* = 0.5498; *MAP1LC3B*: *F*(3,23) = 1.974, *p* = 0.1461; *ATG7*: *F*(3,24) = 11.97, *p* = < 0.0001; *ATG5*: *F*(3,22) = 4.350, *p* = 0.0150; *SQSTM1*: *F*(3,23) = 4.958, *p* = 0.0085; *LAMP1*: *F*(3,24) = 3.429, *p* = 0.0331; ^∗^*p* < 0.05. Error bars show the SEM.

To begin to determine which autophagy associated genes might play a role in HIV-infected subjects with varying levels of NCI, qRT-PCR was used to examine differences in mRNA expression of common genes involved in various stages of the autophagy pathway (*BECN1, MAP1LC3B, ATG7*, and *ATG5*) and those marking autophagic activity (*SQSTM1* and *LAMP1*) from post-mortem brain tissues of the four subject groups [HIV-negative (Group A), HIV-positive (Group B), HIV-positive with NCI (Group C), and HIV-positive with combined NCI and HIVE; Group D)] included in the NNTC Gene Array Project (Gelman et al., 2012). It should be noted that although the Gene Array Project describes samples collected from three brain regions (the frontal lobe white matter, frontal cortex, and basal ganglia), the tissue sample subset that we obtained to analyze consisted almost exclusively of samples from the frontal lobe white matter and frontal cortex (**Table 1**). Coincidently, many of the HIV-infected Gene Array Project subjects had reported histories of polysubstance abuse/dependence (**Table 1**), suggesting that our findings may, in part, be reflective of drug-using populations of infected individuals who could have altered cellular responses from interactive HIV-drug effects occurring in the CNS compared to infected non-substance users (Hauser et al., 2007). Although we did not detect any differences for *BECN1* and *MAP1LC3B* (**Figures 1A,B**), and also for *ATG12* (data not shown), we were able to detect significant differences in *ATG7, ATG5, SQSTM1*, and *LAMP1* expression levels among the subject groups (**Figures 1C–F**). *ATG7* levels were significantly higher in NCI-HIVE subjects compared to all the other groups while *ATG5* levels were significantly lower in HIV-infected subjects with NCI compared to infected subjects without neurocognitive deficits. On the other hand, *SQSTM1* levels had multiple significant differences between the various subject groups including lower expression in HIV-infected subjects that were impaired without HIVE compared to higher expression in NCI-HIVE. Also, *LAMP1* expression levels were significantly higher in HIV-infected subjects that were unimpaired compared to uninfected subjects. Overall, these results suggest that at the transcriptional level while some autophagy-related genes may show differences in expression at various states of HIV infection, the level of autophagic activity may be lower during NCI-HIVE in the frontal lobe white matter and frontal cortex regions of the brain while mixed signaling of activity may occur during HIV infection leading up to NCI.

### Expression Patterns of Autophagy Associated Proteins in HIV-Infected Brain Tissue

**FIGURE 2:**
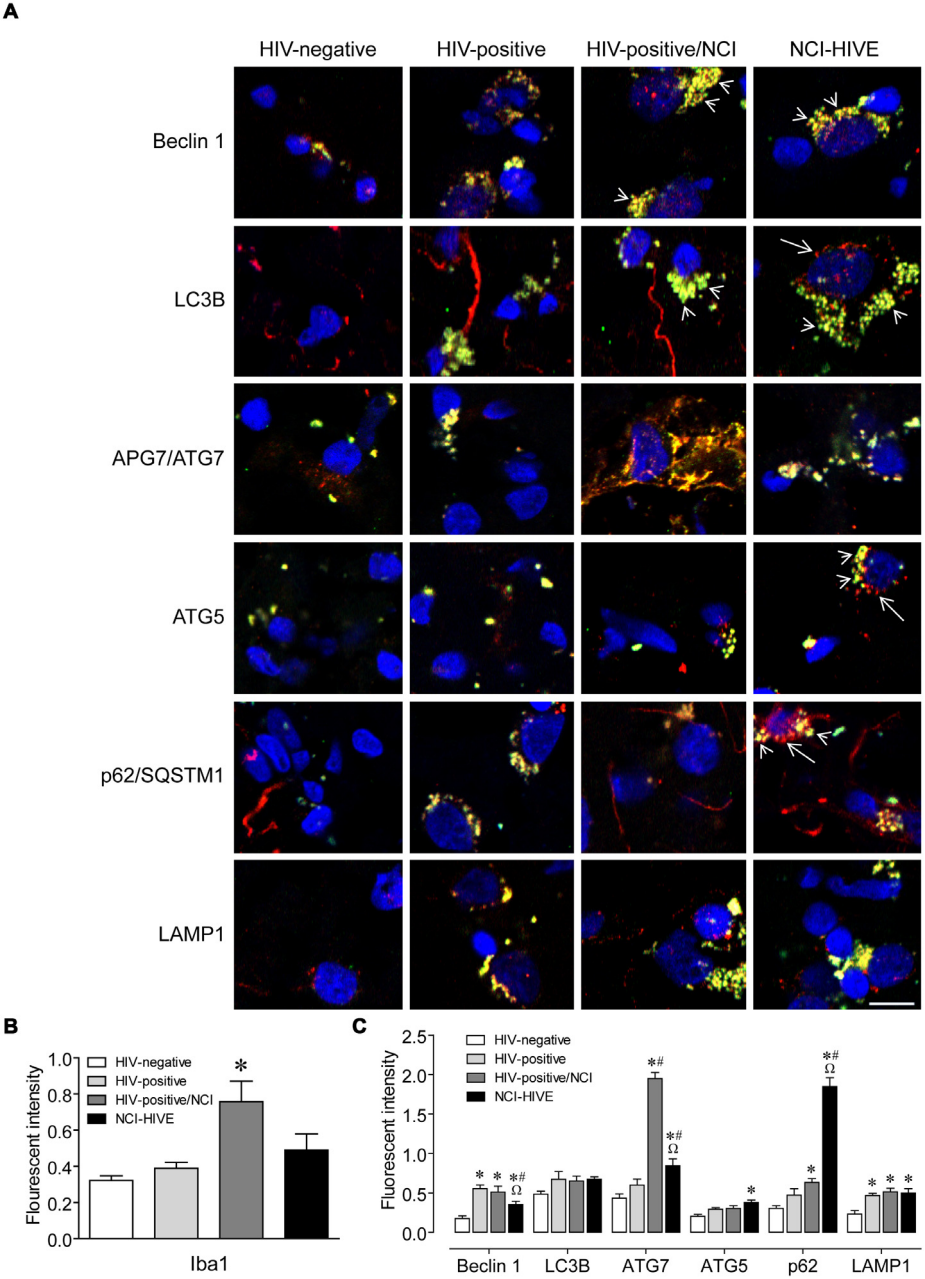
**Autophagy associated protein immunoreactivity in HIV-infected brain tissue. (A)** Representative images from five randomly selected fields of cells each examined in duplicate frontal lobe white matter sections for the indicated subject groups. The indicated proteins were labeled *red* and microglia with the cell-type-specific marker Iba1 (*green*). *Blue* staining indicates cell nuclei. *Arrow heads* indicate examples of higher Iba1 immunoreactivity whereas *arrows* indicate more focal (punctal) vs. diffuse (filamentous) patterns of autophagy associated protein expression. Scale bar = 10 μm. **(B)** Quantification of relative Iba1 immunoreactivity from **(A)**. *F*(3,20) = 6.450, *p* = 0.0031; ^∗^*p* < 0.05 when compared to all other subject groups. Error bars show the SEM for the average values of 2–6 regions from each subject group across the six autophagy associated proteins examined. **(C)** Quantification of the indicated autophagy associated protein relative immunoreactivity from **(A)**. Beclin 1: *F*(3,12) = 11.29, *p* = 0.0008; LC3B: *F*(3,12) = 1.994, *p* = 0.1687; APG7/ATG7: *F*(3,12) = 84.20, *p* = < 0.0001; ATG5: *F*(3,12) = 6.218, *p* = 0.0086; p62/SQSTM1: *F*(3,12) = 87.04, *p* = < 0.0001; LAMP1: *F*(3,12) = 8.317, *p* = 0.0029. ^∗^*p* < 0.05 when compared to HIV-negative; ^#^*p* < 0.05 when compared to HIV-positive; and ^Ω^*p* < 0.05 when compared to HIV-positive/NCI subjects. Error bars show the SEM for four regions from each subject group.

To determine whether there might be differences in protein expression of autophagy associated factors in relation to the qRT-PCR data, we performed immunohistochemistry on representative tissue that was from the same subject samples used in the PCR analysis. White matter sections were used due to the limited amounts of samples available from other brain regions. Autophagy associated protein expression was examined in Iba1-immunolabeled microglia to address whether the differences that we observed might be contributing to microglial-mediated neuroinflammation. Autophagy proteins and related markers were found to co-localize mostly with Iba1-positive microglial cells but were also observed in Iba1-negative cell types (**Figure 2A**). Also, more Iba1-immunoreactive cells were observed in sections from subjects with NCI compared to the unimpaired groups (**Figure 2A**; *arrow heads*). However, when the overall immunofluorescent intensity was quantified, Iba1 expression levels were found to be higher in the NCI group without HIVE (**Figure 2B**). Furthermore, within the Iba1-positive cells from the NCI groups, autophagy associated protein immunoreactivity on individual cells appeared to be higher (**Figure 2A**). When the overall immunofluorescent intensities were quantified for individual autophagy associated proteins, we were able to find significant differences in Beclin 1, APG7/ATG7, ATG5, p62/SQSTM1, and LAMP1 expression levels among the subject groups (**Figure 2C**). Although HIV infection resulted in generally higher expression of LAMP1, and also for Beclin 1 with lower expression in the NCI-HIVE group compared to the other infected groups, higher expression in only the impaired groups was found for APG7/ATG7, ATG5, and p62/SQSTM1. While ATG5 expression was higher only in the NCI-HIVE group, the highest relative levels of expression among the subject groups were found for APG7/ATG7 in the NCI group without HIVE and p62/SQSTM1 in the NCI-HIVE group. Interestingly, more focal rather than diffuse patterns of immunoreactivity were observed among the proteins in the NCI-HIVE group (**Figure 2A**; *arrows*). Although all of these observations did not necessarily correlate with what we detected at the mRNA level, possibly due to post-translational modifications/stability and/or differences occuring specifically in the frontal lobe white matter and/or microglial cell type we examined, these results suggest that at the protein level while higher immunoreactivity may occur in the context of HIV infection and NCI, NCI-HIVE results in the accumulation of autophagy associated proteins in microglial cellular inclusion bodies which could be indicative of altered protein turnover and disruptions to autophagic flux in this cell type and is consistent with what we found for *SQSTM1* by qRT-PCR.

### Examination of Autophagy Associated Genes Differentially Expressed in Various Brain Regions of HIV-Infected Subjects from Microarray Data

**FIGURE 3:**
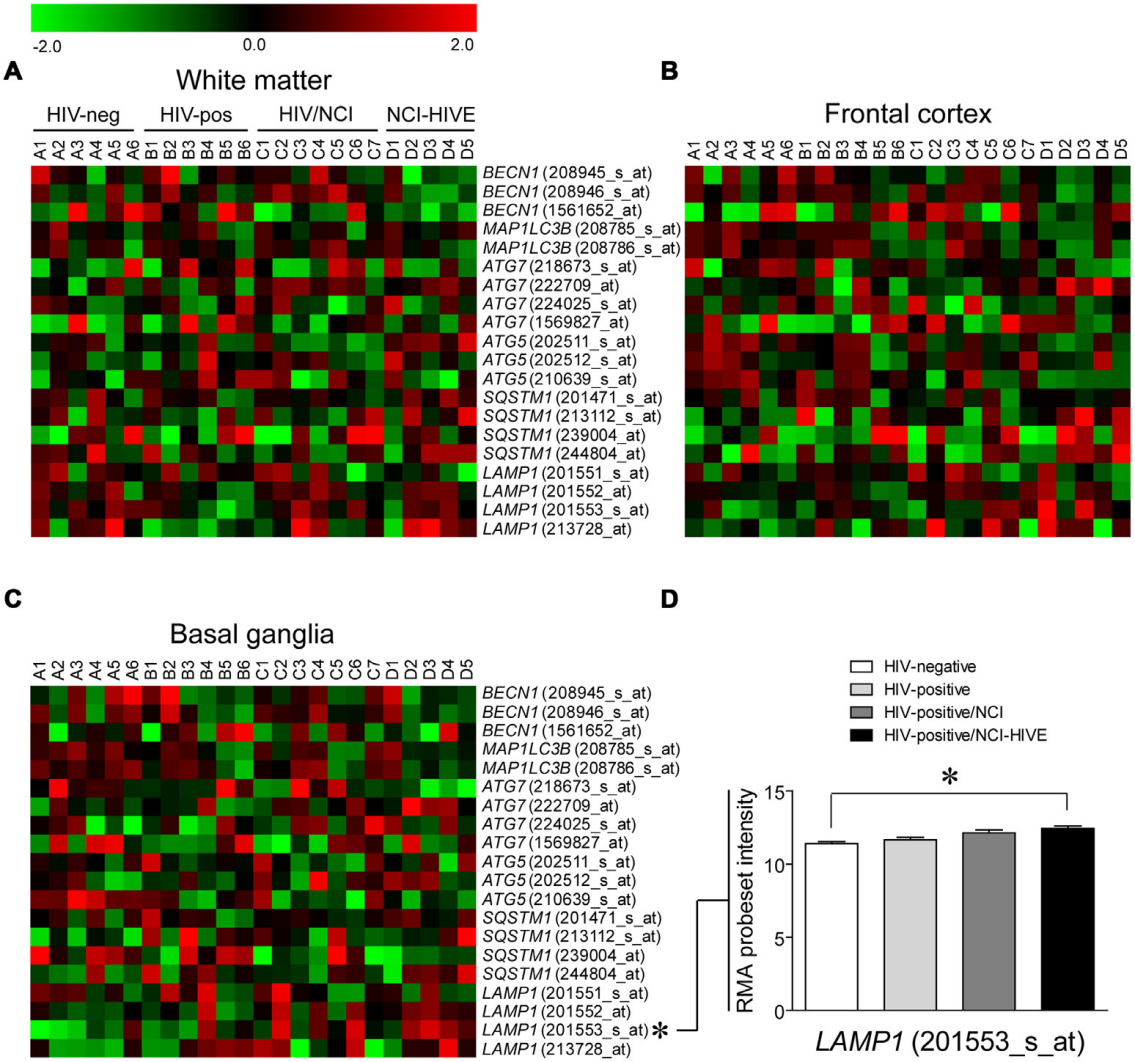
**Autophagy associated gene expression from microarrays in three brain regions of HIV-infected subjects.** Heat maps are shown for the indicated genes from **(A)** frontal lobe white matter, **(B)** frontal cortex, and **(C)** basal ganglia of subjects that were HIV-negative (A1–A6), HIV-positive (B1–B6), HIV-positive with neurocognitive impairment (NCI; C1–C7), and HIV-positive with combined NCI and HIV encephalitis (HIVE; D1–D5). IDs of individual probesets included in the array for each gene are given in parenthesis. **(D)** A significant difference of RMA probeset intensity values from the microarray data analyses was found in the basal ganglia for *LAMP1* (201553_s_at) between HIV-negative (Group A) and NCI-HIVE (Group D) subjects; ^∗^*p* < 0.05. Error bars show the SEM.

Having assayed the subset of brain tissue samples that we had acquired for differences in autophagy associated factors at the mRNA and protein levels by standard methods, we next examined if expression of the genes that were measured by qRT-PCR, as well as whether other autophagy associated genes, might differ among the subject groups in a brain region-specific manner using microarray data that was retrieved and re-analyzed from the same set of subjects used in our study (Gelman et al., 2012; Dever et al., 2014). Microarray data was used for this analysis as all of the tissue samples from individual brain regions included in the Gene Array Project for each subject were not available for qRT-PCR validation experiments, particularly those from the basal ganglia (**Table 1**). Heat maps were generated to display the expression pattern within individual probesets for the genes examined by qRT-PCR across the subject groups from the three brain regions where samples were collected: the frontal lobe white matter, frontal cortex, and basal ganglia (**Figures 3A–C**), and we used a multi-class linear models for microarray data (Limma; parametric) analysis of the microarray data to access differential expression between the subject groups (Smyth, 2004). Out of these genes, only *LAMP1* levels were found to be statistically significant when comparing uninfected and NCI-HIVE subjects in the basal ganglia, which was detected with a particular probeset (201553_s_at; **Figure 3D**), as opposed to the higher levels in HIV-infected subjects without NCI that were detected using qRT-PCR with samples almost exclusively from the frontal lobe white matter and frontal cortex (**Table 1**). This result for *LAMP1* in the basal ganglia was also found in the original microarray analysis of this data set using local-pooled-error (non-parametric) tests for statistical comparisons (Gelman et al., 2012). Although the microarray and qRT-PCR data did not correlate for the genes we had examined up to this point, most likely due to the particular tissue sample subset we used, at the transcriptional level the combined results suggest that autophagic processes may differ in a brain region-specific manner during HIV infection, more closely associated with the NCI-HIVE condition, with higher activity in the basal ganglia as indicated by *LAMP1* levels from the microarray data and lower activity in the frontal lobe white matter and frontal cortex as indicated by *SQSTM1* levels using qRT-PCR. Furthermore, the results from comparison of the genes and corresponding proteins suggest another level of complexity whereby differences in mRNA and protein expression may not necessarily correlate due to modifications/stability at the post-translational level which may also be occurring a brain region- and/or cell-type-specific manner.

In addition to the genes examined thus far, we also wanted to determine whether our Limma analysis found differences with probesets for other autophagy associated genes as well. The analysis found that the autophagy-related gene *ATG4D*, as well as other genes involved in autophagy including *DRAM1* and *KIAA0226*, had significantly differential expression between uninfected and NCI-HIVE subjects in the basal ganglia, and *ATG12* and *ATG13* as well as *CTSB, TM9SF1*, and *WIPI1* levels significantly differed when all the subject groups were compared in this same brain region, although no significant pair-wise differences were found between any two particular groups (data not shown). Also, *ATG3* and *ATG4D* as well as *C12orf44* (*ATG101*), *AMBRA1, DRAM1, KIAA0226, RAB24*, and *ULK1* in the frontal lobe white matter and *DAP* and *DRAM1* in the frontal cortex were found to have significant differences across all groups (data not shown). The differential expression of these other genes that we did not examine by qRT-PCR might represent interesting targets for future validation studies on the differences that occur in autophagy during various states of HIV infection, and, in particular, NCI-HIVE. Furthermore, these results suggest that the basal ganglia, where most of the microarray differences were found, may be a key brain region to evaluate the impact of differing autophagic processes on the neurocognitive function of HIV-infected patients.

### Effect of Supernatant from HIV-1-Infected Microglia on Neuronal Autophagic Activity in the Presence of Morphine

**FIGURE 4:**
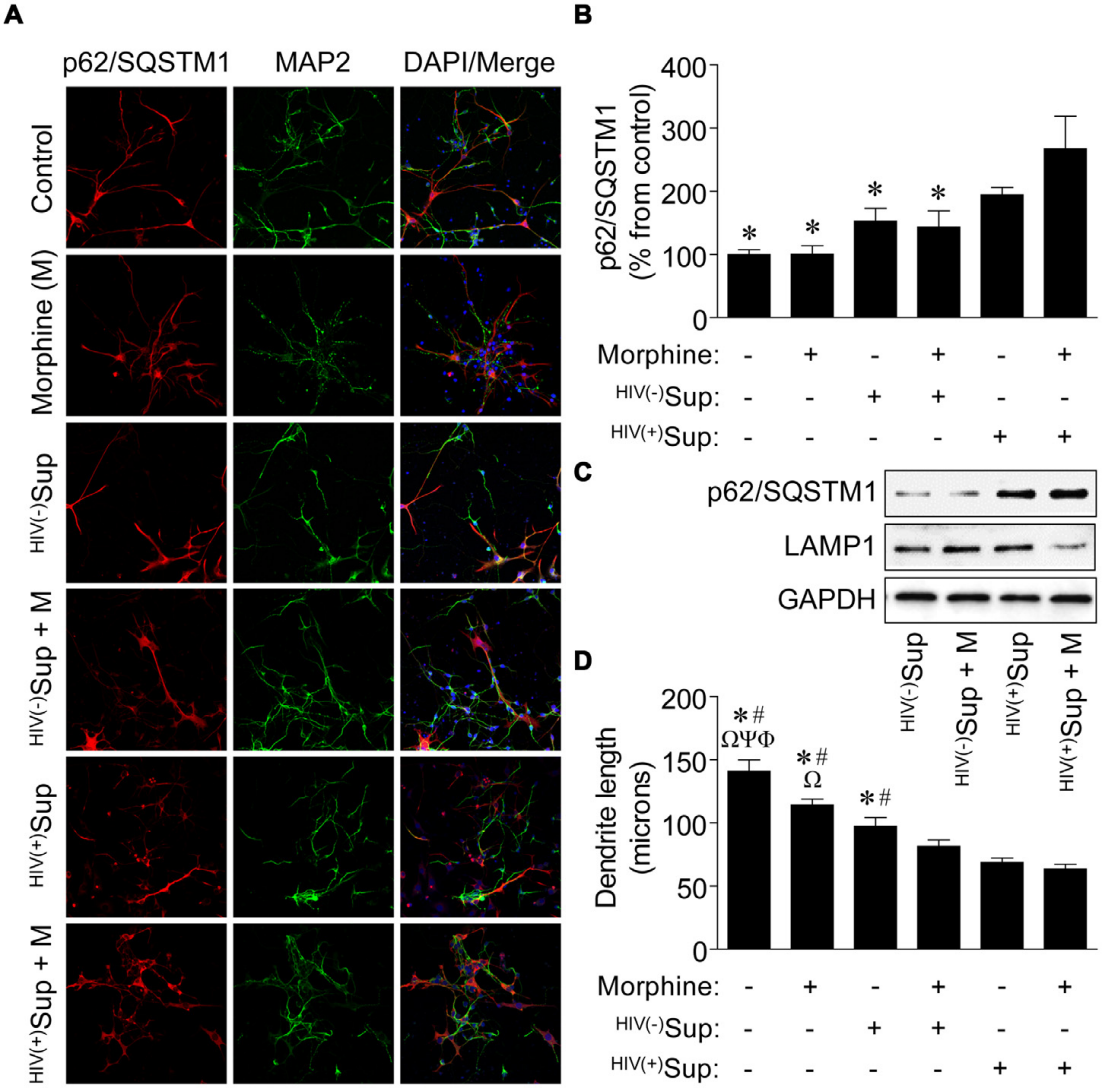
**Effects on autophagic activity and dendritic length of neurons exposed to supernatant from HIV-1-infected microglia in combination with morphine. (A)** Representative images of neurons with the indicated treatments. Sup, supernatant from uninfected [HIV(-)] and HIV-1-infected [HIV(+)] microglia. Cells were immunolabeled with antibodies to the autophagic activity marker p62/SQSTM1 (*red*) and the neuronal cell-type-specific marker MAP2 (*green*). DAPI (*blue*) staining indicates cell nuclei. **(B)** Quantification of p62/SQSTM1 immunoreactivity from **(A)**. Data are presented as the percentage of control cells which was set at 100; *F*(5,24) = 5.882, *p* = 0.0011; ^∗^*p* < 0.05 when compared to ^HIV(+)^Sup + morphine treatment. **(C)** Western blotting analysis of p62/SQSTM1 and LAMP1 expression levels for the indicated treatments. GAPDH was used as a loading control. Blots are representative of three independent experiments. **(D)** Measurement of dendrite length from **(A)**. *F*(5,24) = 26.15, *p* = < 0.0001; ^Φ^*p* < 0.05 when compared to morphine; ^Ψ^*p* < 0.05 when compared to ^HIV(-)^Sup; ^Ω^*p* < 0.05 when compared to ^HIV(-)^Sup + morphine; ^#^*p* < 0.05 when compared to ^HIV(+)^Sup; and ^∗^*p* < 0.05 when compared to ^HIV(+)^Sup + morphine treatment. Error bars show the SEM for five randomly selected fields totaling at least 100 cells from each group.

As disruptions to the autophagic activity of primary rodent neurons have been reported to result with treatment of supernatant from SIV-infected microglia cultured *ex vivo* (Alirezaei et al., 2008), and HIV-1-infected primary human microglia exposed to morphine *in vitro* can affect autophagy which may influence the downstream neurotoxic activities of this cell type (El-Hage et al., 2015), we next tested whether *in vitro* exposure of primary human neurons to supernatant from HIV-1-infected primary human microglia in the presence of morphine might also modify autophagic processes in a manner that could result in altered neuronal function leading to NCI. Although many of the HIV-infected Gene Array Project subjects had substance use histories with various drugs of abuse (**Table 1**), we chose to examine the interactive effects of HIV and morphine on neurons as opiates with abuse liability that preferentially activate the μ-opioid receptor (MOR) such as morphine can exacerbate the neuropathogenesis of HIV by mechanisms which are not fully understood (Hauser et al., 2005). However, it is generally believed glia play a central role in mediating opiate drug-HIV interactions on neurons through bystander events via neurotoxic signaling molecules and viral proteins released from infected and activated cells such as microglia and that opiates such as morphine can potentiate these effects (Hauser et al., 2012). Interestingly, while the supernatant from HIV-1-infected microglia alone did not result in differences to autophagic activity as determined by measuring p62/SQSTM1 immunoreactivity in exposed neurons, supernatant from infected microglia in the presence of morphine resulted in significantly higher p62/SQSTM1 expression (**Figures 4A,B**), suggesting inhibition of autophagy as we found for NCI-HIVE subjects *in vivo*. In support of these observations, when whole-cell lysates were examined by western blotting analysis, we found that p62/SQSTM1 expression levels were highest for infected supernatant and combined morphine treatment compared to the other supernatant treatments, and which corresponded with concomitantly lower LAMP1 levels (**Figure 4C**). Also, when the length of neuronal dendrites was examined under these same conditions, we observed that morphine alone was able to reduce dendritic length which was further enhanced in the combined presence of supernatant from uninfected microglia, and even more so in combination with supernatant from HIV-1-infected microglia, demonstrating an additive trend in response to the supernatant and morphine treatments (**Figure 4D**). These results suggest that differences in dendritic length can occur in response to morphine and HIV-1-infected microglial supernatant alone rather than as a consequence of inhibited autophagic activity which was only observed with co-treatment. Furthermore, anatomical variations might occur in a transient/reversible manner to initial exposure possibly resulting in milder forms of NCI, while severe NCI may correlate with structural differences coupled to mechanistic alterations in cellular function through inhibition of autophagic activity that could lead to more permanent neurocognitive deficits and dementia as with NCI-HIVE subjects.

### Influence of HIV-1 Tat and Morphine Treatment on Autophagy in Neurons

**FIGURE 5:**
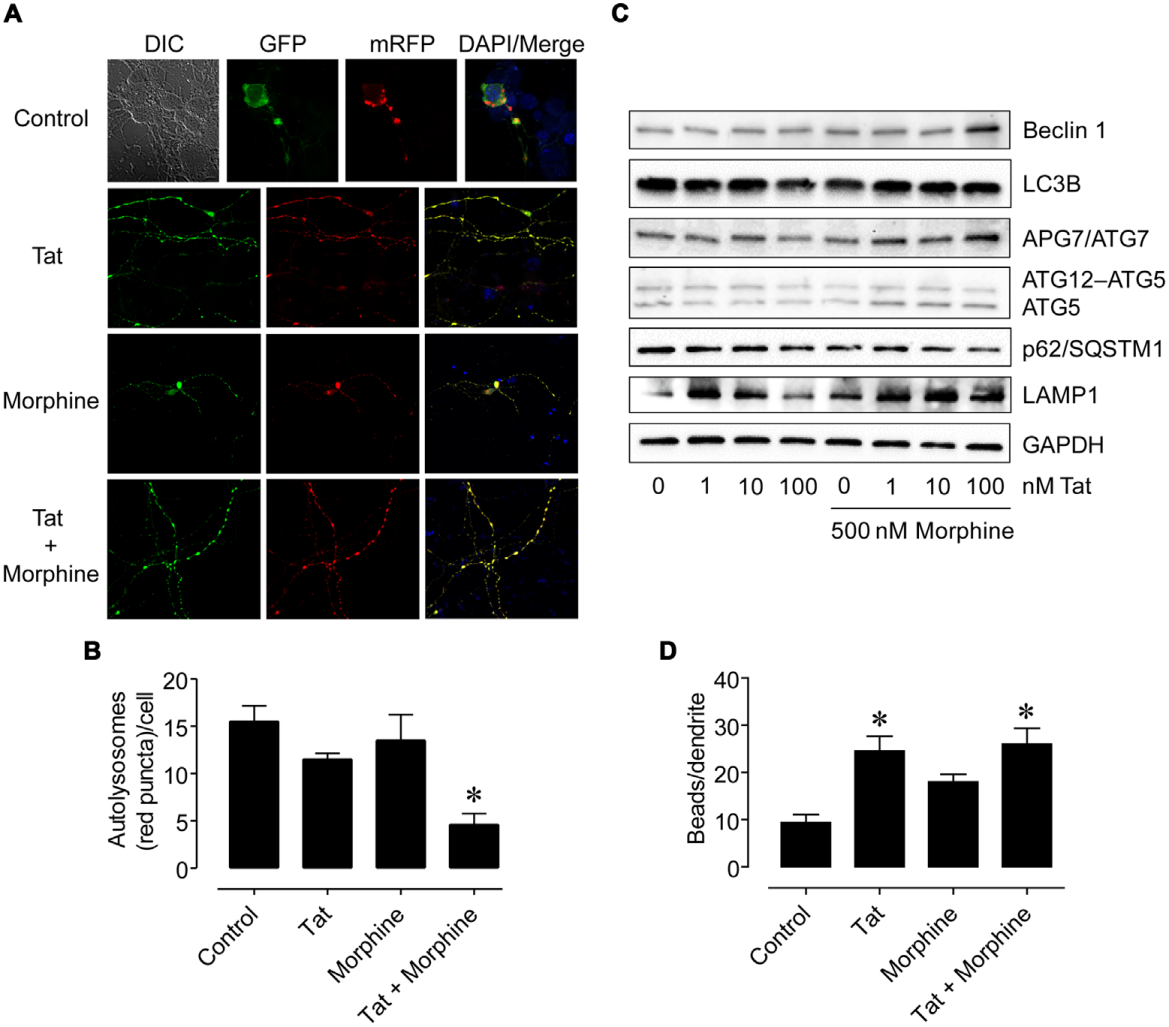
**Differences in neuronal autophagy and dendrite varicosity following HIV-1 Tat protein and morphine treatment. (A)** Representative images of neurons transfected with a fluorescent reporter plasmid to monitor autophagic flux at 8 h following the indicated treatments. GFP (*green*) and GFP + mRFP (*yellow*) fluorescence are observed prior to the fusion of autophagosomes with lysosomes whereas only mRFP (*red*) fluorescence is present in post-fusion autolysosomes. *DIC*, differential interference contrast microscopy image. DAPI (*blue*) staining indicates cell nuclei. **(B)** Quantification of autolysosomes (*red* puncta) from **(A)**. *F*(3,13) = 8.756, *p* = 0.0019; ^∗^*p* < 0.05 when compared to all other groups. **(C)** Western blotting analysis of the indicated autophagy associated protein levels at 24 h following the indicated treatments. GAPDH was used as a loading control. Blots are representative of three independent experiments. **(D)** Quantification of dendrite beading from **(A)**. *F*(3,77) = 6.429, *p* = 0.0006; ^∗^*p* < 0.05 when compared to control cells. Error bars show the SEM.

Since the HIV-1 Tat protein can mediate neuronal toxicity which can be enhanced in the combined presence of morphine (Zou et al., 2011), we then tested if treatment of primary human neurons with HIV-1 Tat and morphine would have similar effects to what we found with the supernatant from HIV-1-infected microglia and morphine to further delineate whether various cellular-oriented factors such as inflammatory molecules released by infected microglia or viral proteins shed from these cells may be responsible for the interactive effects by first using a fluorescent reporter system to monitor autophagic flux. Neurons were transfected with a plasmid encoding a mRFP-GFP-LC3 tandem fluorescently-tagged LC3 reporter with which green (GFP) and yellow (GFP + mRFP) fluorescence are observed prior to the fusion of autophagosomes with lysosomes whereas only red (mRFP) fluorescence is present in post-fusion autolysosomes (Kimura et al., 2007). Following transfection, neurons were treated with HIV-1 Tat [an initial concentration of 100 nM was tested which can elicit functional deficits in neurons and glia similar to those occurring in HIV infection and is considered to reflect levels seen pathophysiologically (Nath et al., 1999; Singh et al., 2004; El-Hage et al., 2005, 2008)] and morphine [500 nM was chosen for the ability of this concentration to fully activate MOR and synergistically enhance Tat-mediated neurotoxicity (Gurwell et al., 2001; Zou et al., 2011)] alone or in combination for 8 h and the differential patterns of fluorescence were observed (**Figure 5A**). When the number of red fluorescent signals (puncta) occurring in autolysosomes were manually quantified, we found that HIV-1 Tat and morphine treatment in combination resulted in significantly lower numbers of autophagosomal–lysosomal fusion events compared to all the other treatments (**Figure 5B**). However, when whole-cell lysates were next examined by western blotting analysis for expression levels of various autophagy associated proteins at a later time point (24 h), autophagic activity appeared to be higher. While LC3B levels remained relatively constant across the treatment conditions and LAMP1 levels were higher for a lower concentration of Tat (1 nM) which was sustained using higher concentrations of Tat (10 and 100 nM) in the presence of morphine, Beclin 1, APG7/ATG7, and ATG5 exhibited higher levels of expression coinciding with combined Tat and morphine exposure, that were mostly associated with the highest concentration of Tat used (100 nM), when p62/SQSTM1 levels were concomitantly lower (**Figure 5C**). We also observed that dendritic beading, which is indicative of excitotoxic stress and/or injury (Ellis et al., 2007), was significantly higher with HIV-1 Tat treatment and Tat treatment in combination with morphine compared to control cells (**Figure 5D**). All combined, our *in vitro* results suggest that while HIV infection can result in bystander effects on neuronal morphology, disruptions to specific cellular mechanisms such as autophagy may occur more readily from the interactive effects of HIV and associated viral proteins with substances of abuse/dependence such as morphine. Furthermore, when morphine is present, the consequences of neurotoxic and inflammatory factors released by infected cells on neuronal autophagic processes may be different than that of viral proteins which could in themselves have differing effects in a time-dependent manner.

## Discussion

In the present study, we investigated the role of autophagy in the brains of HIV-infected subjects with NCI with and without combined HIVE. These findings are unique in that our subject group categories allowed us to determine whether the differences we detected in autophagy may be more associated with encephalitis than NCI. For instance, this seemed to be the case when we examined *SQSTM1* expression using qRT-PCR, and our results were in agreement with a previous report that found higher mRNA levels of this gene in the frontal lobe of SIV encephalitic (SIVE) monkeys and frontal cortex of human subjects with HAD (Alirezaei et al., 2008). It should be noted that most of our subjects with NCI had neurocognitive diagnoses of MCMD and possible HAD, while those with NCI-HIVE were diagnosed with HAD and probable HAD (**Table 1**).

We began our search for differences in autophagy associated factors by performing qRT-PCR and immunohistochemical analyses on brain tissue samples obtained from various categories of HIV-infected subjects, followed by examining microarray data generated from these same subjects within three brain regions. Interestingly, out of several genes commonly examined in autophagy studies that we also investigated, the microarray data only revealed a significant difference in expression for the autophagic activity marker *LAMP1* which was detected with a particular probeset in the basal ganglia. However, we were able to detect significant expression differences among the subject groups for other genes involved in autophagy as well using qRT-PCR. In addition, the difference found for *LAMP1* in the microarray data was between uninfected and NCI-HIVE subjects whereas the difference detected by qRT-PCR was between uninfected and HIV-infected subjects without NCI. The discrepancy in these results could be at least partially explained by the particular tissue sample subset provided to us which consisted almost exclusively of samples from the frontal lobe white matter and frontal cortex (**Table 1**), as all of the samples used in the arrays were not available. This limitation points to the importance and need for brain banking and availability of well-matched samples from different anatomical regions and disease state categories of HIV-infected subjects for future validation studies.

Furthermore, we found differences with our immunohistochemical analysis of autophagy associated proteins in microglia that were not reflected in the qRT-PCR data suggesting differential regulation at the transcriptional, translational, and post-translational levels. For example, the immunoreactivity of proteins that we examined was generally higher in the impaired groups. However, our results for p62/*SQSTM1* were fairly consistent by both qRT-PCR and immunohistochemistry with higher expression in the NCI-HIVE group. Overall, our findings of immunoreactivity were mostly in agreement with a previous report that found higher expression levels for autophagy associated proteins in the frontal cortex of HIVE individuals by western blotting analysis (Zhou et al., 2011). Interestingly, Zhou et al. (2011) reported that no differences in the protein expression levels were found between HIVE-positive individuals with and without HAD. Combined with our findings, the data again suggest that encephalitis more than NCI alone may be responsible for some of the observed differences. Although we were able to observe differences in immunoreactivity using tissue sections, we were unable to perform other assays to confirm these findings due to the limited amount of tissue available. The availability of larger tissue sample sizes from the categories of subjects that we examined will be useful to further evaluate our findings at the protein level by more quantitative methods such as western blotting.

In addition to differences in immunoreactivity, we also found qualitative differences in the expression of autophagy associated proteins with more focal rather than diffuse patterns in microglia from the NCI-HIVE group. This finding suggests that in the HIVE condition, accumulation of proteins associated with autophagy in cellular inclusion bodies may be indicative of altered protein turnover, which could be related to what we observed as higher levels of immunoreactivity, and is manifested by disruptions in autophagic activity/flux. Our results are supported by a previous report that found more focal patterns of p62/SQSTM1 expression in the hippocampus of monkeys with SIVE and HIV-infected individuals with HAD (Alirezaei et al., 2008). Indeed, such formations have been described in patients with a variety of neurodegenerative diseases (Zatloukal et al., 2002). Furthermore, *SQSTM1* transcription can be higher as part of inclusion body formation (Nakaso et al., 2004), which supports our qualitative and quantitative (p62/SQSTM1) immunohistochemical results with the qRT-PCR data for this gene that we detected in the NCI-HIVE group. Overall, our findings with the human brain tissues suggest HIVE, rather than NCI alone, is more associated with differences to autophagic processes in microglia which may then through altered intercellular transmission of released factors result in similar differences within neuronal cells that could lead to neurodegeneration, and p62/SQSTM1 is a responsive marker in this regard. Future *in vitro* studies will be needed to systematically explore the connections between disrupted autophagy in microglia and the implications for autophagic and other cellular processes in bystander cell types such as neurons.

While our human brain tissue data was fairly consistent with the findings of both Alirezaei et al. (2008) and Zhou et al. (2011), the combined results from these and our study seem somewhat contradictory suggesting that autophagy levels are both higher and lower during HAD/HIVE. However, it may be possible to reconcile these findings in that induced or suppressed autophagic activity in response to prolonged HIV infection to, for example, clear toxic factors and affect viral replication, followed by compensatory differences to offset these effects itself suggests a mechanism whereby mixed signals and dysregulation in an attempt for cell survival is manifested as cellular dysfunction which could lead to NCI. Interestingly, a more recent study found that non-progressor HIV-infected patients have higher autophagy compared to normal progressors when peripheral blood mononuclear cells (PBMCs) were examined (Nardacci et al., 2014). This finding suggests that, at least in the periphery, higher autophagy levels may be critical for controlling the initial spread of HIV infection to the CNS, preventing the formation of viral reservoirs in the brain which could eventually lead to HAD and HIVE. Future studies will be needed to evaluate the connections between the higher and lower autophagic activity to identify targets which could possibly be used to offset and/or coordinate the opposing events.

In conjunction with our human *in vivo* data, we also found that morphine can interact with the supernatant from HIV-1-infected microglia and HIV-1 Tat protein in opposing ways to cause differences of neuronal autophagy *in vitro*. These results suggest that opioid exposure may uniquely activate some of the *in vivo* effects of HIV through modulation of the autophagic pathway in this cell type which could accelerate NCI compared to bystander events from HIV infection alone. Furthermore, these mechanistic differences resulting from interactive effects could potentially result in more permanent alterations to cellular function compared to the morphological differences that we observed in dendritic length and beading, which may be reversible (Ellis et al., 2007), with individual treatments. However, as a common route of HIV infection in substance-abusing individuals is following injection drug use (Leshner, 1998), the consequences of preexisting and irreversible dendrite differences from chronic drug exposure that can itself result in NCI may be exacerbated upon the onset and duration of HIV infection with more additive rather than interactive effects in this sequence of events leading to further neuronal injury and death.

Interestingly, while our *in vivo* human brain tissue data correlated with that of both Alirezaei et al. (2008) and Zhou et al. (2011), our *in vitro* results did not as a significant effect of supernatant from HIV-1-infected primary human microglia and Tat treatment alone on autophagy in primary human neurons was not found for the most part. Alirezaei et al. (2008) demonstrated that supernatant from SIV-infected microglia cultured *ex vivo* inhibited autophagy in primary rodent neurons, whereas Zhou et al. (2011) showed HIV-1 gp120 protein exposure resulted in higher autophagic activity of SK-N-SH neuroblastoma cells. However, our results in conjunction with morphine on autophagy did agree with both Alirezaei et al. (2008) and Zhou et al. (2011) showing induction with viral protein and inhibition for infected microglial supernatant following 24 h of treatment, while the interactive effect with Tat appeared to be dynamic with lower autophagic activity at 8 h co-exposure. Although the effects on autophagy that we were able to find compared to Alirezaei et al. (2008) and Zhou et al. (2011) may have resulted from exacerbation by morphine and could also be attributed to variations in the species, SIV/HIV strains, HIV proteins, cell systems, etc. used between all three studies, future characterization of supernatant from infected microglial cells and testing the effect of individual components on autophagic processes in neurons will be necessary for understanding the underlying differences.

Furthermore, recently published *in vitro* data from our lab supports the *in vivo* findings of this study that showed HIV-1 infection can affect autophagy in primary human microglia and morphine can modulate these differences which could influence viral replication, cytokine/chemokine production and release, and potentially other factors involved in mediating neurotoxicity (El-Hage et al., 2015). Therefore, future evaluation of the *in vitro* effects from morphine and other drugs of abuse on autophagy in the context of HIV infection examining individual CNS cell types may provide new insights for particular therapeutics directed toward the autophagic pathway and which target an individual type of cell. However, at present, the interactions between HIV and autophagy are largely unknown in astrocytes (Dinkins et al., 2015), and other CNS cell types such as pericytes and brain microvascular endothelial cells involved in blood-brain barrier integrity. Elucidation of the basic mechanisms by which HIV infection affects autophagy in these cells will be necessary before detailed studies on the impact of drugs in these processes can be systematically explored. As astrocytes are thought to be important for driving HIV-opiate interactions through intercellular feedback loops with microglia (Hauser et al., 2012), their role in autophagy during HIV infection may represent a potential therapeutic strategy targeting the bystander effects of glial responses on neurons and worth intense future investigation. In addition, detailed studies on a single drug may not be feasible using *in vivo* human samples, and particularly with our tissues, as many of the infected subjects from our study had a history of polysubstance abuse/dependence (**Table 1**). This constraint further underscores the notion that availability of tissue samples from HIV-infected subjects with well-documented patient histories of a particular substance use, as well as those with no reported use history, will be necessary for future evaluation when using *in vivo* human samples to examine an individual drug and also highlights the need for more *in vitro* studies on the interactive effects from exposure to multiple drugs of abuse in the context of HIV infection.

## Conclusion

Our findings demonstrating differences of autophagy in microglial cells from NCI-HIVE subjects are one of the few reports that suggest microglia may represent a better upstream therapeutic target to modulate autophagy in the context of HIV infection to ameliorate neurodegeneration rather than neuronal cells themselves. However, for HIV-infected individuals exposed to morphine, direct neuronal targeting may be as important as targeting glia. Therefore, substance-abusing individuals may require more aggressive and multifaceted treatment regimens targeting the direct and indirect cellular causes of neurocomplications from HIV infection than non-substance users.

## Author Contributions

SD and NE-H designed the study and analyzed and interpreted the experimental data. SD, MR, JL, and NE-H conducted the experiments. BC retrieved and analyzed the microarray data. SD drafted the manuscript and NE-H critically revised the work for important intellectual content. All authors approved the final version of the manuscript.

## Conflict of Interest Statement

The authors declare that the research was conducted in the absence of any commercial or financial relationships that could be construed as a potential conflict of interest.

## Footnotes

1 http://www.ncbi.nlm.nih.gov/geo/

2 http://www.tm4.org/mev.html
